# Preliminary results of using ALAnerv® in subacute motor incomplete paraplegics

**Published:** 2015

**Authors:** I Andone, A Anghelescu, C Daia, G Onose

**Affiliations:** * ”Bagdasar Arseni” Teaching Emergency Hospital (BATEH), Bucharest, Romania; ”Carol Davila” University of Medicine and Pharmacy, Bucharest, Romania

**Keywords:** motor incomplete paraplegia, neuroprotection, neurotrophic compound, nutritional supplement

## Abstract

**Rationale:** To assess whether using ALAnerv® contributes to improvements of outcomes obtained in post SCI patients.

**Objective:** A prospective controlled clinical survey also to evaluate the safety and efficacy of ALAnerv® (2cps/ day for 28 days) in motor incomplete (AIS/ Frankel C) paraplegic subacute patients.

**Methods and results:** 59 patients divided in study (treated with ALAnerv®) and control, groups. This survey’s follow-up duration was of 28 days. Most of the studied patients were mid-aged (mean 43.75 years old) and respectively, men (64,29% in the study group; 58,06% in controls). We used descriptive statistics (functions: minimum, maximum, mean, median, standard deviation) and for related comparisons, parametric (Student t) and non-parametric (Mann-Whitney, Fisher’s exact, chi-square) tests.

The primary end-point: AIS motor values’ evolution (P= 0.015 - Mann-Whitney), showed that patients treated with ALAnerv® – vs. controls – had a statistically significant better increase of such scores at discharge. Paraclinical parameters, mainly exploring systemic inflammatory status (secondary end-point): ESR dynamics (P=0.13) had no statistical significance; the plasma leucocytes number (P=0.018), the neutrophils’ percentage (P=0.001) and fibrinogenemia (P= 0,017) proved in the treated group to have a statistically significant better evolution. We used “Statistical Package for Social Sciences” (SPSS).

**Discussion:** As there is actually no effective curative solution for the devastating pathology following SCI, any medical approach susceptible to bring even limited improvements, such as treatment with ALAnerv® seemed to have proven, is worth being surveyed, under strict circumstances of ethics and research methodology. Considering the necessity for more statistical power concerning primary, secondary end-points, and safety issues, as well, continuing this research is needed.

**Abbreviations:** SCI = spinal cord injury, TSCI = traumatic spinal cord injury, BBB = blood brain barrier, CNS = central nervous system, SC = spinal cord, NSAIDs = non-steroidal anti-inflammatory drugs, SAIDs = steroidal anti-inflammatory drugs, AIS = American Spinal Injury Association Impairment Scale, SPSS = Statistical Package for Social Sciences, BATEH = Bagdasar-Arseni Teaching Emergency Hospital

## Introduction

Because Spinal Cord Injury (SCI) is a condition that frequently generates severe and rather permanent disabilities [**1**-**4**], most of them with no cure, many therapeutic directions are oriented to neuroprotection. So, ALAnerv® (produced by Alfa Wassermann SpA Italy), being a nutritional supplement with relatively well-known, determined, neurotrophic effects on other – peripheral [**5**-**11**] - neurological conditions, but having all its main components acceptably passing through the Blood Brain Barrier (BBB) [**12**-**15**], we decided to develop a study on post SCI patients, with motor incomplete paraplegia, in subacute stage, to assess whether - and if so, to what extent – the use of this complex compound might contribute to some improvement of their neuromotor/ functional outcome.

Regarding the possible benefits of ALAnerv® in post-traumatic spinal cord injury (TSCI) patients, there are quite encouraging, but only few, experimental, studies (on animals) with its components [**12**,**16**,**17**]. 

What should be emphasized is that although disc hernias and spinal channel stenosis can be found in literature – together with the possible causes of SCI, but classified as degenerative conditions [**18**] - we considered these (also taking into account our very consistent, included, long term expertise in the management of SCI) to have, in fact, micro-traumatic intimate path-physiological mechanisms and therefore, we decided to include them among the conditions matching the inclusion criteria for this survey.

**Fig. 1:**
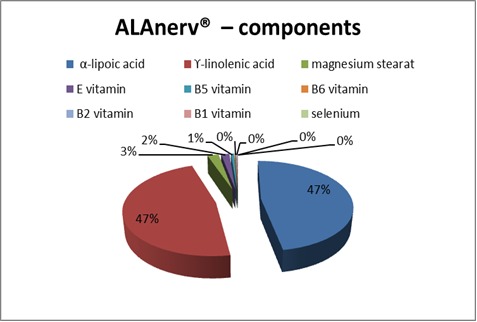
ALAnerv® – weighted graphic representation by its composition

ALAnerv® – nutrition supplement/ OTC already long time registered and approved to be marketed - has in its composition, a complex of substances, with different and complementary roles. Hence, such a gelatinous capsule, of 972,807 mg has, as main active compounds: 300 mg α-lipoic acid, lamb-tongue - Borago officinalis containing 22% γ-linolenic acid (300mg; fatty acids triglycerides 60 mg) -, 15 mg magnesium stearate, natural E vitamin 1000UI/ g (D-α-tocopherol on sunflower oil support), poliglicerol oleate - 10 mg complex of soya oil and lecithin from soya -, 5,396 mg calcium D-pantothenate - equivalent to 4,5 mg B5 vitamin -, 2,010 mg pyridoxine-chlorhydrate - equivalent to 1,5 mg B6 vitamin -, 1,320 mg ribovlavine - equivalent to 1,2 mg B2 vitamin -, 1,259 mg thiamine mononitrate - equivalent to 1,05 mg B1 vitamin -, 55 mcg anhydrous sodium selenite - equivalent to 25 mcg selenium) (**Fig. 1**).

This compound is (we considered this, too) quite well balanced regarding the molecules it entails and their related doses. In this respect there are quite consistent data [**9**,**12**,**19**-**24**] in literature, but which, unfortunately, cannot be detailed in this article because of its – naturally - limited editorial space.

**Study design**

This was a prospective controlled clinical survey, realized in respect to the guidelines exposed in the Declaration of Helsinki, performed in the Physical (neural-muscular) and Rehabilitation Medicine [P(n-m)RM] Clinic Division of “Bagdasar-Arseni” Teaching Emergency Hospital (BATEH), approved by the Bio-Ethics Commission of this hospital and by the Alimentary Bioresources Institute, subordinated to the Ministry of Agriculture and Rural Development – appointed by the regulations to authorize the entrance on the market of ALAnerv® nutritional supplement.

The aim was to assess the safety and efficacy of ALAnerv® administration - with a medium duration of 4 weeks (28 days according to prospectus: inference from peripheral nerve pathology) - in motor incomplete (AIS/ Frankel C – according to the worldwide largely accepted/ used related classification of post SCI neurological/ functional status, endorsed by the American Spinal Cord Injury Association – ASIA – and the International Spinal Cord Society - ISCoS [**25**] paraplegic subacute patients who sustained a thoracic and/ or lumbar TSCI (including micro-trauma – as afore commented) within the first 2 months of initial injury, by comparing the related clinical functional evolution/ results (at baseline and at the end of the treatment), during their first hospitalization in our Clinic, to those obtained in a similar kind of patients, but who did not receive this nutritional supplement or other neurotrophic compounds, but only standard therapy and rehabilitation.

**The study group** - treated with ALAnerv® - was established prospectively starting from October 2012 (the survey is still ongoing), based on written informed consent, obtained from each patient, previous to his/ her enrollment in the study.

**The control group** was constituted – taking into account ethical considerations regarding the equality of chances - retrospectively (data extracted from inpatient medical records, starting with January 2009, untill the end of the study, for patients who, for different objective reasons, have not been included/ will not be included in the study group), based on the same inclusion and exclusion criteria as the lot treated with ALAnerv®. 

**Inclusion criteria:** age ≥ 18 years; subacute TSCI (less than 2 months elapsed since the initial lesion - including microtrauma); motor incomplete spinal cord impairment - AIS/ Frankel C; paraplegia (injury level below T1).

**Exclusion criteria:** age < 18 years, more than 2 months elapsed since the initial TSCI, other spinal cord lesion (AIS/ Frankel) than C; spinal cord lesion level above T2; spinal cord pathology with other etiology than TSCI (infection, tumor, autoimmune diseases/ multiple sclerosis, etc.); preexistent peripheral nerve disorders of non-(micro-/) traumatic etiology; severe orthopedic trauma possibly interfering with recovery and/ or appropriate neuromotor/ functional testing; any cerebral pathology (vascular disease/ stroke, tumor, autoimmune disease/ multiple sclerosis, infection, dementia of any type, trauma - except for initially mild, and remitted at the moment of admission in our NeuroRehabilitation ward); neoplasm of any kind/ organ, in any stage; heart/lung/liver/kidney failure(s); refusal of the patient to be enrolled in the study.

**Treatments:** All the patients in the study group received ALAnerv®, 2 capsules per day, administered just before eating/ at the beginning of the meals, in the morning and at noon, for 28 consecutive days, according to the product profile and prospectus.

All the patients received the necessary and appropriate complex treatment, including rehabilitation, for their condition and co-morbidities, in accordance with current guidelines: antithrombotics/ anticoagulants, antibiotics, urinary antiseptics, pain killers, non-steroidal anti-inflammatory drugs (NSAIDs), steroidal anti-inflammatory drugs (SAIDs), intravenous fluids, mucolytics – each if necessary. 

Even if, accordingly to our study concept, NSAIDs and SAIDs should be listed as not allowed medications, some pain and inflammatory local phenomena, determined their administration, for patients’ benefit – usually sporadically and on short time – thus being admitted among allowed drugs. Therefore, it can be asserted, regarding possible statistical afferent significances related to the dynamics of some inflammation markers’ – secondary end-point – this issue to be a possible, but ethically justified, limitation of the study (see further).

All the studied patients have followed aside an adapted rehabilitation/ care program: kinesiotherapy, including assistive devices; bladder/ bowel training/ management; somatic cover integrity prophylaxis and/ or care; psychotherapy, if necessary.

Not allowed medication - as it might interfere with the post TSCI neurological outcome -: other products with neuroprotective and/ or neurotrophic effects (any component of ALAnerv®, Pyrithinol, Cerebrolysin, nucleosides - Keltican, Nucleo/ Forte), psychotropic drugs.

**Primary endpoint:** to quantify the improvement - if existing - of the AIS scores (motor, sensory) by comparing them to the ones achieved in the control group, over the same period of time. 

**Secondary endpoints:** speed of the neuromotor/ functional rehabilitation process, using the “days until the first knee functional extension” as evaluation parameter: quadriceps muscle force ≥ 3; parameters reflecting the evolution of the inflammatory status (erythrocytes sedimentation rate –ESR - mm/ h - , fibrinogenemia – mg/ dl -, plasma leucocytes number - /mm3 -, neutrophils percentage %). 

All the patients treated with ALAnerv® were informed about the potential side effects related to this therapy and asked to promptly report any adverse event they observe (nausea, vomiting, abdominal pain, abnormal or worsened hepatic tests, allergies, sleep disturbances, bleeding and/ or abnormal coagulation tests in patients receiving concomitant antithrombotic or anticoagulant therapy, etc.).

## Methods

We have surveyed 59 patients, divided in a study – treated with ALAnerv® - group (28 cases) and a control one (31 patients). Both groups were assessed in a unitary mode, at baseline and at the end of the first admission. The patients were followed-up over a mean period of 4 weeks: 39.68 days, median 35.50 (for the treated group) and respectively, 35.55 days, median 34.00 days (for the controls).

Their evaluation was based on epidemiological data, clinical AIS scores, para-clinic items (see above) and based on the measurement of the respective parameters, we have evaluated the treatment responsiveness. 

Statistical data were processed in SPSS, Microsoft Excel 2010. To compare differences in parametric data we used the Student t - when parameter values had a normal distribution - and the Mann-Whitney test when we have not found such of population distribution normality. Fisher’s exact test and chi-square tests were employed to compare non-parametric (frequency) data. All values were considered of significant difference if related P<0,05 [**26**,**27**].

## Results

The main statistical characteristics of lots, especially mean, median and standard deviation (std. dev.) for all the important parameters in both groups are detailed in **Table 1**.

**Table 1:** Mean, median and standard deviation (std. dev.) for all the important parameters in both groups

Type of treatment	ALAnerv® treated lot			Control lot		
Parameter	Mean	Std. Dev.	Median	Mean	Std. Dev.	Median
Age	43.7	17.33	39	43.8	13.80	45
Hospitalization days	39.7	19.21	35.5	35.6	13.07	34
AIS Motor Score at admission	69.1	6.45	72	67.4	6.65	69
AIS Motor Score at discharge	79.5	11.25	82	74.3	10.90	74
Difference of AIS Motor scores	10.5	7.08	9.5	6.9	7.08	4
AIS Sensitive Score at admission	199.6	20.98	196	202.1	23.13	204
AIS Sensitive Score at discharge	213.0	12.33	218	204.6	22.49	204
Difference of AIS Sensitive scores	13.4	14.58	10	2.5	5.94	0
ESR at admission	63.6	31.56	66.5	74.3	43.12	72
ESR at discharge	28.0	18.94	23	41.7	27.52	35
Difference of ESR values (admission/ discharge)	35.6	30.97	29	32.6	41.84	8
Fibrinogenemia at admission	640.7	153.48	675	640.4	161.2	650
Fibrinogenemia at discharge	449.1	124.69	445.5	529.3	16.84	530
Difference of Fibrinogenemia (admission/ discharge)	191.6	147.38	203	111.2	135.85	59
Plasma Leucocytes number at admission	10.8	13.69	8.195	8.2	2.70	7.5
Plasma Leucocytes number at discharge	8.0	8.67	5.9	7.477	3.50	6.4
Difference of Plasma Leucocytes number (admission/ discharge)	2.8	5.90	2.2	0.8	2.23	1
Neutrophils at admission (%)	63.4	13.92	66.9	63.8	10.79	63.7
Neutrophils at discharge (%)	53.9	14.46	55.4	60.7	11.71	60.7
Difference of neutrophils (%- admission/ discharge)	9.5	12.82	8.85	3.1	7.29	3.4
Number of days until LEFT knee first extension	20.3	22.30	12.5	27.6	24.41	18
Number of days until RIGHT knee first extension	22.3	23.53	12.5	23.6	23.74	15

**Fig. 2:**
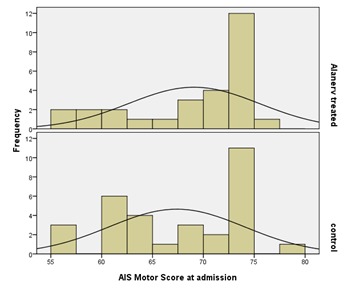
Frequency histograms of the AIS Motor Scores at admission

The initial comparison of the groups was done mainly in relation to motor AIS scores at admission and because of the sensible differences between specific means and medians, in order to work on sampling as adequately as possible, to enable the process of compatible comparative inputs, we eliminated 3 patients from the control group; the related histograms showing the scores values are represented in **Fig. 2**.

Comparing the lots by the nonparametric Mann-Whitney test (preferred because it is difficult to accept the assumption of normality plots data – see **Fig. 2**), a P value of 0.325, associated with the statement that groups differ, resulted. So we could not exclude the fact the lots were similar in terms of AIS motor scores at admission.

**Fig. 3:**
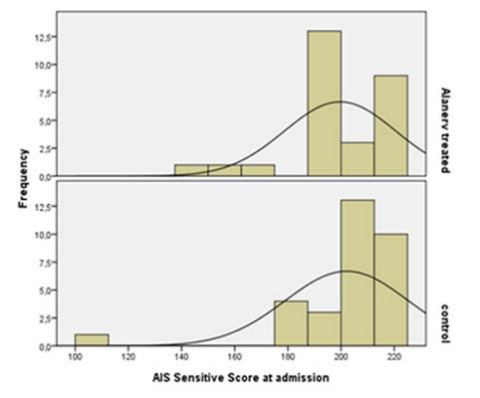
Frequency histograms of the AIS Sensitive Scores at admission

Regarding the AIS sensitive scores using the same non-parametric test (P= 0,531) associated with the affirmation that the groups differ between them, we could not exclude the fact that the lots were similar from the point of view of AIS sensitive scores at admission (**Fig. 3**).

**Fig. 4:**
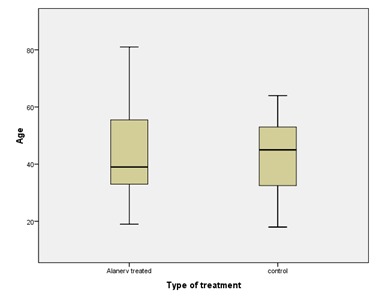
Age distribution plot boxes in both study and control groups

From the point of view of age at admission, we could observe that the mean were very close (43.68, respectively 43.81 years), but a discrepancy appeared between the medians (39, respectively 45 years), reflecting the fact that the patients treated with ALAnerv® were younger, this being clearly showed in **Fig. 4**.

**Fig. 5:**
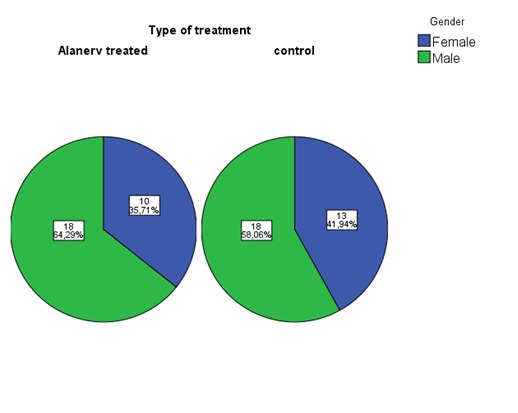
The distribution by gender

The groups’ structure by gender was statistically similar (P=0.790) by exact Fisher test (preferable because of the small number of patients in the groups) and most of the patients were men: 64,29% in the study group; 58,06% in controls (**Fig. 5**).

**Fig. 6:**
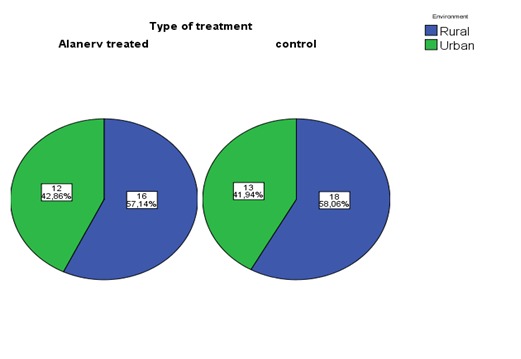
Distribution by residence/ provenance

Regarding the residence distribution, using the exact Fisher test (P= 1.0) indicated similar distribution, most of the cases having rural provenance: 57,14% in study group and 58,06% in controls (**Fig. 6**).

**Fig. 7:**
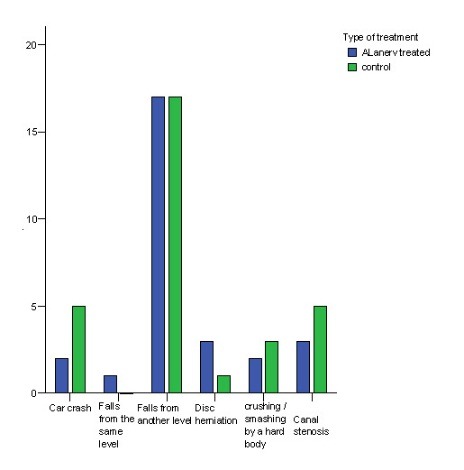
Causes of SCI

The most frequent SCI cause in both groups (60,71% in the study lot, and 54,83% in the controls) was falling from other level (**Fig. 7**); we could not exclude the similarity of distribution by SCI causes but neither the dissimilarity (P=0.572 - chi-square test).

**Fig. 8:**
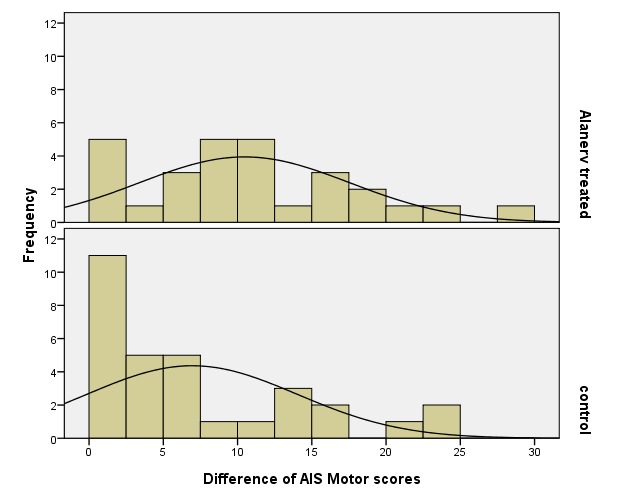
Histograms regarding the evolution of AIS Motor scores

The “Efficacy” of the treatment with ALAnerv® could be shown by the evolution (AIS Motor score at discharge – AIS Motor score at admission) of AIS motor score from the two groups. The bigger the difference mentioned above, the “better” the patient evolved. Histograms from the differences of scores are represented in **Fig. 8**. The mean in the ALAnerv® study group showed an improvement of AIS Motor score with 10.5 units and, in the control group, only with 6.9 units. Also, the median of the score differences was of 9.5 units in the group treated with ALAnerv® , and only 4 units in the control group. These data allowed us to make the affirmation that the treatment with ALAnerv® had a positive effect on patients (P=0.015 - Mann-Whitney test), improving AIS Motor score (better than in the absence of the treatment) and represented statistical significance of those data.

**Fig. 9:**
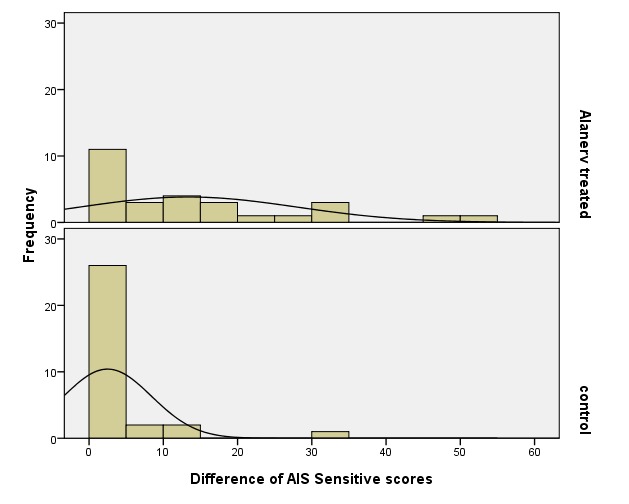
Histograms regarding the evolution of AIS Sensitive scores

The evolution of AIS Sensitive scores (AIS Sensitive score at discharge – AIS Sensitive score at admission) are represented in **Fig. 9**. In mean, the study group improved its AIS Sensitive score with 13.4 units and the control group with only 2.5 units. The median of score differences was of 10 units in the study lot and only 0 units for controls. Those data allowed us to make the affirmation that the treatment with ALAnerv® had a positive effect on patients (P =0.001 - Mann-Whitney test), significantly improving their AIS Sensitive score (more than in the absence of the treatment).

**Fig. 10:**
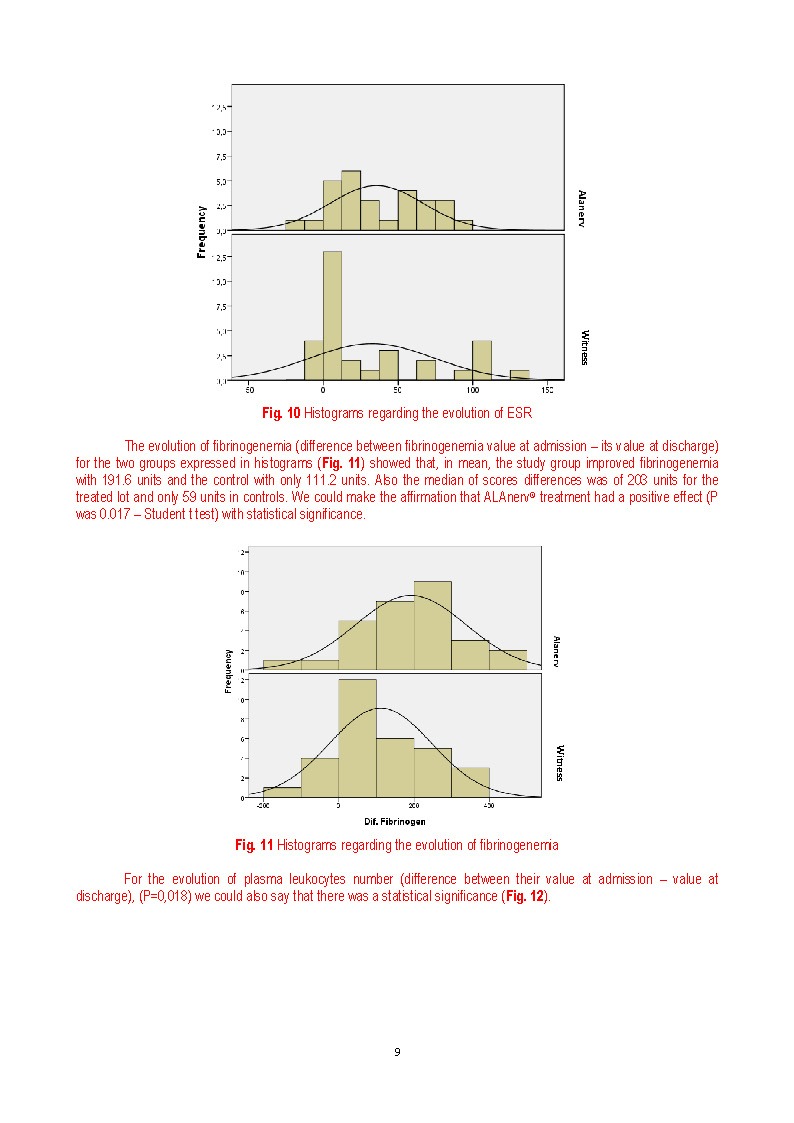
Histograms regarding the evolution of ESR

The evolution of ESR (difference between ESR value at admission – ESR value at discharge) expressed in histograms (**Fig. 10**) showed, in mean, that the study group improved ESR with 35.6 units, the median being of 29 units and the control one with mean of 32.6 units, the median being of only 8 units. Those data allowed us to make the affirmation that ALAnerv® treatment had a positive effect, also improving ESR values. However, because the P value was of 0.13, i.e. much above 0.05 threshold, the difference detected had no statistical significance.

**Fig. 11:**
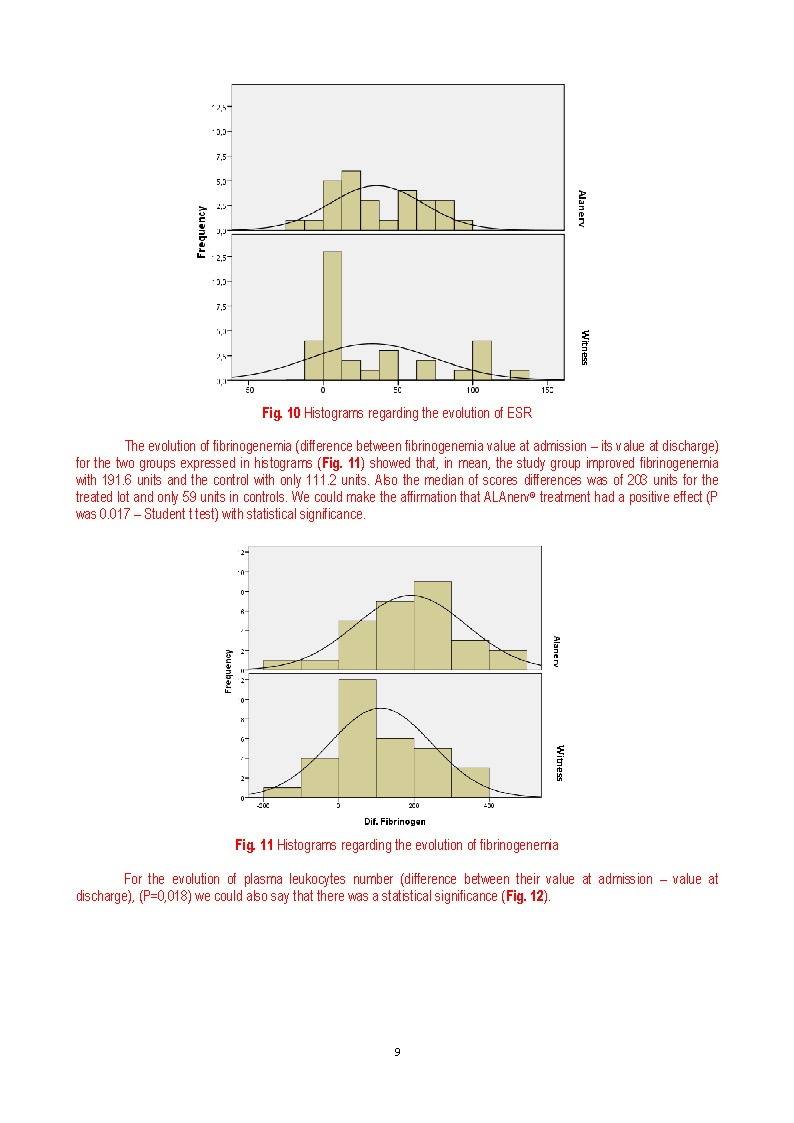
Histograms regarding the evolution of fibrinogenemia

The evolution of fibrinogenemia (difference between fibrinogenemia value at admission – its value at discharge) for the two groups expressed in histograms (**Fig. 11**) showed that, in mean, the study group improved fibrinogenemia with 191.6 units and the control with only 111.2 units. Also the median of scores differences was of 203 units for the treated lot and only 59 units in controls. We could make the affirmation that ALAnerv® treatment had a positive effect (P was 0.017 – Student t test) with statistical significance.

**Fig. 12:**
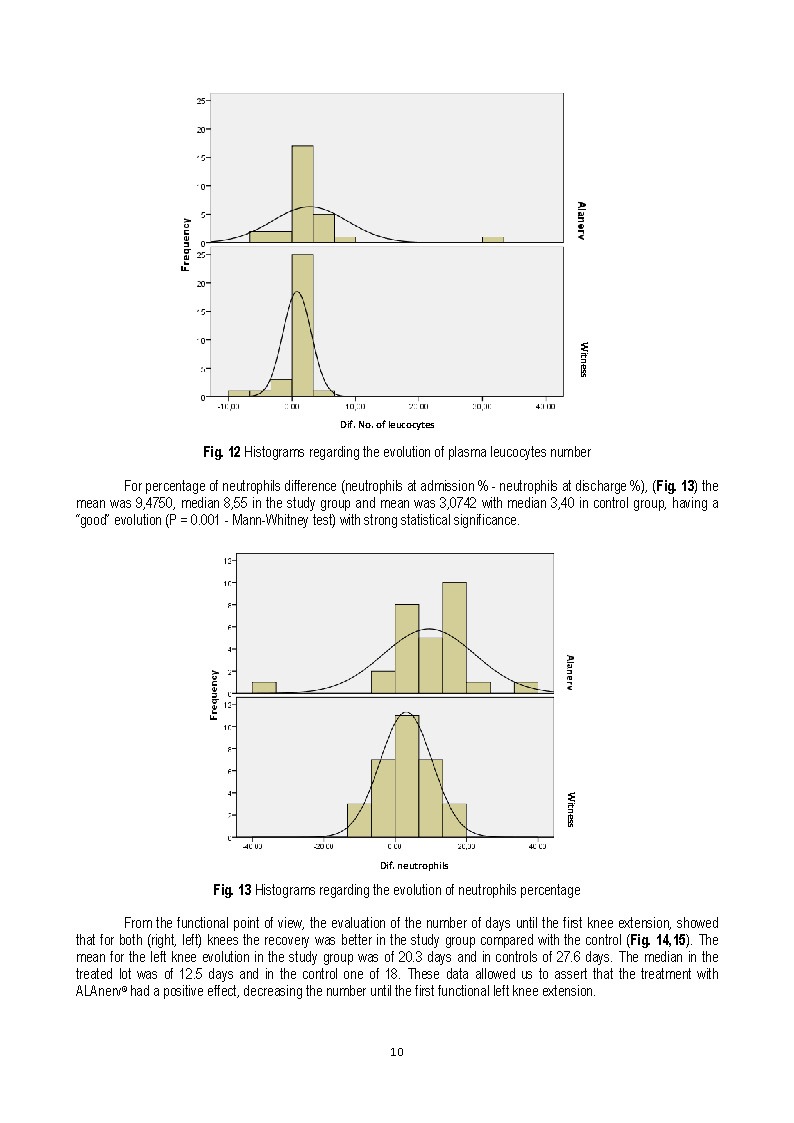
Histograms regarding the evolution of plasma leucocytes number

For the evolution of plasma leukocytes number (difference between their value at admission – value at discharge), (P=0,018) we could also say that there was a statistical significance (**Fig. 12**).

**Fig. 13:**
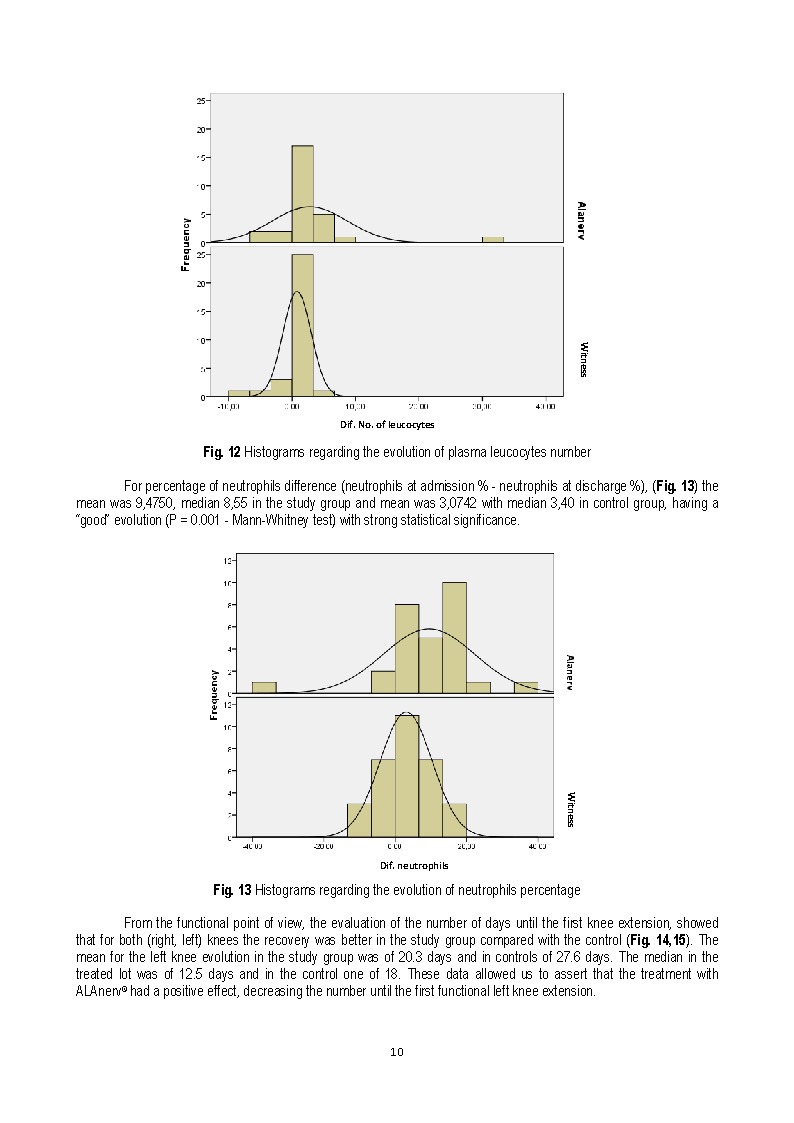
Histograms regarding the evolution of neutrophils percentage

For percentage of neutrophils difference (neutrophils at admission % - neutrophils at discharge %), (**Fig. 13**) the mean was 9,4750, median 8,55 in the study group and mean was 3,0742 with median 3,40 in control group, having a “good” evolution (P = 0.001 - Mann-Whitney test) with strong statistical significance.

**Fig. 14:**
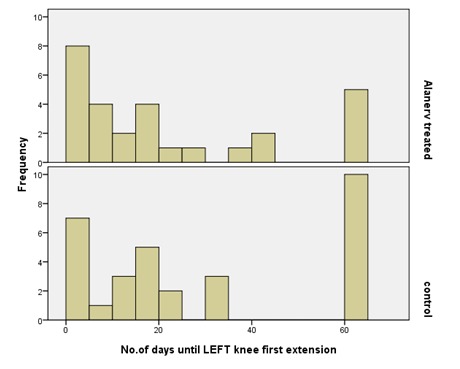
No. of days until Left knee first extension evolution histogram

**Fig. 15:**
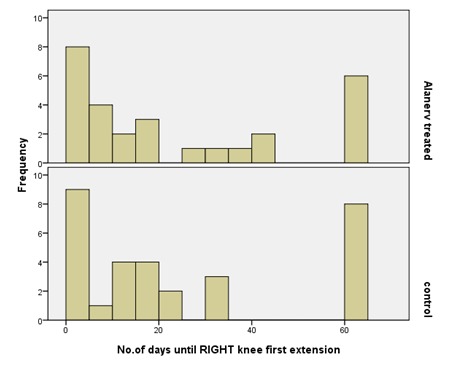
No. of days until Right knee first extension evolution histogram

From the functional point of view, the evaluation of the number of days until the first knee extension, showed that for both (right, left) knees the recovery was better in the study group compared with the control (**Fig. 14**,**15**). The mean for the left knee evolution in the study group was of 20.3 days and in controls of 27.6 days. The median in the treated lot was of 12.5 days and in the control one of 18. These data allowed us to assert that the treatment with ALAnerv® had a positive effect, decreasing the number until the first functional left knee extension.

As for the right knee, the mean was of 22.3 days in study group and 23.6 days in controls, and median of 12.5 days in the treated, compared to 15 days on controls. Treatment with ALAnerv® had a positive effect on it, too, but (P=0.751 -Mann-Whitney test) with no statistical significance. Therefore, from this point of view, the ALAnerv® treatment did not show any statistically significant improvement.

## Discussion

The preliminary results of the survey objectify the possibility that by using ALAnerv® to obtain a significant improvement of the neuromotor and sensitive/ functional outcomes and also to decrease the levels of fibrinogenemia, plasma leucocytes number and neutrophils percentage, but with no statistical significance for the ESR dynamics. Accordingly, a limitation of the present study refers to the markers of inflammation that were used (commonly available in our ward): insufficiently to establish a definite conclusion regarding (also) an anti-inflammatory effect of ALAnerv®; additionally the connected limitation regarding sporadic/ by necessity use of NSAIDs/ SAIDs in some patients has already been mentioned before.

Another limitation of this study: post SCI patients - including incomplete motor paraplegics in subacute stage - were, during such a targeted period like that of our study (both regarding its retrospective and prospective dimensions) in spinal shock - the first 3 of 4 phases of this phenomenon last over the first 4 (possibly 6) weeks - thereby subject to a component of spontaneous recovery, although this did not linearly relate with the voluntary motor control regain, as the spinal shock’s hallmark was (only) ”the transient suppression and then the gradual return of reflex activity, caudal to SCI” [**28**]. 

Not to mention that it is reasonably presumed that the spontaneous recovery might occur in both lots (ALAnerv® treated and controls); so, it could be considered to the significantly better outcomes obtained in the study group, the treatment with ALAnerv® could contribute to make the difference, especially as the follow-up duration of the survey – and implicitly the short duration, for a neurotrophic agent, on which the effects of ALAnerv® have been traced - also enabling the assumption that this compound might also hasten a related functional recovery.

On the other hand, it has to be honestly emphasized that the targeted category of patients: AIS/ Frankel C had been chosen as the most prone stage of this kind of pathology to be both: subjects of an objective improvement (unlike the AIS B and especially AIS A ones) and without major risks for a ceiling effect – as we recon, could be possible for the AIS D.

As possible side effects in the study group, we observed three cases of transitory moderate elevated liver transaminases and one with a greenish (biliary?) asymptomatic stool (not repeated and not clearly related to the treatment). The liver tests were corrected/ normalized after stopping the whole treatment, this being a problem to be further investigated (whether such transitory abnormalities could be exclusively attributed to ALAnerv® or not).

The disease approached in this study, generally with no cure, remains a complicate, difficult medical challenge, with considerable disabling potential thus being a public health matter. Therefore, any possible, even modest beneficial (but with no significant adverse) effects, are worth studying and, if safe and effective, clinically valorized. 

**Acknowledgements**

Many thanks to the Company Alfa Wassermann S.R.L. Romania for providing the nutritional supplement ALAnerv® used in this study. This survey has been done without money funding.

**Disclosures**

The providers of ALAnerv® (Alfa Wassermann S.R.L. Romania) are old and constant partners of our societies (the Romanian Society for NeuroRehabilitation and the Romanian Spinal Cord Society) and Clinic Division. They contributed to the support of scientific meetings, training courses and/ or participation of members, with accepted papers, to congresses/ conferences/ symposia, but they did not interfere in any way with this study (data collection or processing, concluding results or editing endeavors). 
